# Reproducibility of pop sensation, Thompson sign in achillotomy, and final Pirani score to predict clubfoot relapse: Achillotomy clinical signs and Pirani predictive ability

**DOI:** 10.1097/MD.0000000000038377

**Published:** 2024-06-14

**Authors:** Sergio Charles-Lozoya, Héctor Cobos-Aguilar, Jorge Luis Alvarado-Alanis, Miguel Leonardo De la Parra-Márquez, Arnoldo Salas-Delgado, Marcela Araceli Segoviano-Mendoza, Héctor Eliud Arriaga-Cazares, Jocelyn Verónica Montes-Cruz

**Affiliations:** a Health and Research Science Management, Pediatric Orthopedic Surgery, Division of Plastic and Reconstructive Surgery, Hospital de Traumatología y Ortopedia No. 21, Instituto Mexicano del Seguro Social (IMSS), Monterrey, NL, Mexico; b Health Science Division, Vice-rectory of Health Science, Universidad de Monterrey, San Pedro Garza Garcia, NL, Mexico; c Coordination of the Doctorate of Medical Sciences, Universidad Juárez del Estado de Durango, Durango, Durango, Mexico; d Monterrey Regional Hospital ISSSTE, Monterrey, NL, Mexico.

**Keywords:** achilles tendon, diagnostic ultrasound, reproducibility, talipes equinovarus, tests

## Abstract

Ultrasound (US) can guide and confirm percutaneous release of the achilles tendon in the clubfoot. However, this technique may not always be available; therefore, surgeons’ reported feelings of tendon release (“click” or “pop”) and the Thompson sign could demonstrate that they are sensitive and reliable for confirming complete tendon release. The purpose of this study was to compare the reproducibility of clinical maneuvers that aim to detect the reported “click” or “pop” sensation by the surgeon and the Thompson sign after surgical release in percutaneous achilles tenotomy compare with US in patients with clubfoot. A cross-sectional reproducibility study of consecutive patients with idiopathic clubfoot was conducted. All the patients were scheduled to undergo tenotomy in the operating room using the standard percutaneous achilles tenotomy technique under sedation. The surgeon’s reported surgical sensation (“click” or “pop”) and Thompson signs were compared to the US assessment of the cut. The final Pirani score was used to predict recurrence risk and was correlated with the number of plaster casts and age. Forty-five feet were affected in 30 patients. Eighteen (60%) men. Age range: 1 to 60 months. The sensation of “click” or “pop” was recorded in 38 patients, and complete release was confirmed by US in 37 patients, for a sensitivity (Se) of 0.95 and specificity (Sp) of 0.63. Thompson signs were positive in 33 and 36 patients at 2 evaluations, with Se values of 0.87 and 0.92 and Sp values of 0.88 and 0.75, respectively. The Pirani final score, a predictor of recurrence risk, had an area under the curve of 0.80 (95% CI = 0.63–0.97; *P* = .005), Se = 0.78, and Sp = 0.56, with a cutoff point of 2.75. The feeling of achilles tendon release and Thompson sign had high sensitivity, prevalence, accuracy, and posttest probability. The confirmation of tendon release based on clinical signs could prevent the use of US.

## 1. Introduction

Idiopathic congenital talipes equinovarus (ICTEV), also known as clubfoot, is a common congenital defect with a prevalence between 0.4 and 6.8 per 1000 births in Chinese, Caucasian, and Polynesian populations.^[1–3]^ This condition has ethnic, etiological, and clinical genetic variations and could be associated with environmental and intrauterine factors.^[4]^ Treatment of ICTEV requires orthopedic intervention via the Ponseti method,^[5]^ which involves weekly manipulations with plaster casts and percutaneous achilles tenotomy (PAT). PAT is necessary in 80% of patients because of the shortening and stiffness of the tendon, which limits joint movement in the ankle.^[6]^ PAT is considered an outpatient surgery performed with local anesthesia and has minimal risk; however, the inflammation caused by local anesthetics makes it difficult to palpate the achilles tendon (AT), which can result in a partial incision, risk of recurrence,^[7]^ iatrogenic injuries to the posterior tibial neurovascular package, peroneal artery, saphenous vein, and sural nerve.^[8]^ Therefore, some surgeons and those with limited experience in percutaneous methods prefer to perform open AT tenotomy with a small incision under general anesthesia to visualize the tendon and achieve complete cutting.^[9,10]^

On the other hand, ultrasonography (US) is a useful method for evaluating tendons because it can reveal their dimensions, morphology, and texture.^[11]^ It is also reliable for diagnosing AT rupture, with a sensitivity (Se) of 0.94 to 0.99 and specificity (Sp) of 0.98 to 0.99.^[12]^ Additionally, it is a noninvasive and dynamic tool because it allows real-time visualization of tendons and can be used to control cuts “in vivo.” It is accessible, repeatable, simple to perform, and can be used in outpatient settings.^[13]^ For these reasons, it is advantageous to guide PAT in the treatment of ICTEV, as well as for diagnostic performance, by demonstrating in real time that the AT has been completely sectioned, which could reduce the recurrence of equinus.^[14]^

However, to avoid incomplete cuts of AT in the PAT technique and if US is not available, it is crucial to estimate the diagnostic capacity of clinical maneuvers to diagnose a complete AT cutoff because repeated maneuvers and movements may risk the posterior tibial package.^[15]^ Among the clinical signs that could be indicators of complete AT sectioning are the surgeon’s report of a “click” or “pop” sensation; however, this sign in the US analysis, has a low Se of 0.44 for complete AT sectioning,^[16]^ while the Thompson sign refers a Se and Sp of 0.96 and 0.93, respectively.^[17–19]^ There have been no studies on the use of either sign as a test reproducibility for AT cuts in patients undergoing PAT.

In contrast, the Pirani scale, which is predominantly used to assess the progression of ICTEV deformity correction, is also employed to predict recurrence based on the initial evaluation score.^[20]^ However, the prognostic value of the final total Pirani score has not been explored prior to PAT, but it can be concluded that the final score, as well as the subcomponent score for the hindfoot, are independent predictors of early recurrence at 6 months.^[21]^

Therefore, the main objective of this study was to estimate the reproducibility of clinical maneuvers that aim to compare US revision with “click” or “pop” sensation feeling reported by the surgeon and the Thompson sign after surgical release in PAT in patients with ICTEV and, secondarily, to analyze the ability of the final Pirani score to predict the presence of recurrence.

## 2. Materials and methods

A cross-sectional test reproducibility study was conducted between 2022 and 2023 in patients with ICTEV who underwent PAT at Hospital No. 21 for Orthopedic and Trauma Surgery in northeastern Mexico, which is part of the Mexican Social Security Institute. The protocol was approved by the ethics committees of the Research and Health Research Local Committees (registration number *R*-2019-1903-008). Informed consent was obtained from the parents of the patients enrolled in the study. The study included patients with idiopathic clubfoot who, after manipulation using Ponseti method, exhibited dorsal flexion ≥‐10° or a Pirani score ≤1 for the midfoot and ≥1 for the hindfoot^[22]^ (see below for descriptions of both). Patients who had a previous PAT, were treated with open tenotomy, or had complex or syndromic clubfoot were excluded from the study.

### 2.1. Selection and sample size

Study participants were selected via non-probabilistic sampling of consecutive patients from the surgical registry. To calculate the sample size, the statistical software Epidat 4.2 was used for diagnostic tests in paired groups based on the report by Karami et al.^[16]^ The “click” or “pop” sensation of percutaneous tendon cutting was managed with a Se = 0.80 and for US = 0.99 (12), with a 95% confidence level, resulting in a total of 43 feet.

### 2.2. Data collection and procedures

Patients were treated according to the Ponseti method,^[23–25]^ which involves manipulating the cavus deformity, followed by maintaining foot abduction beneath the talus head and correcting all deformity elements simultaneously, except for equinus, which was addressed through PAT. Plaster cast was padding and molding, commenced at the heel and extended to the thigh Deformity correction was evaluated using the Pirani scale, which has an interobserver kappa of 0.63 to 0.80^[26,27]^ and considers visual aspects (medial and posterior crease, curvature of the lateral border), palpation (coverage of the talus and empty heel), and functional aspects (rigidity equinus). Each sign was scored 0 (normal), 0.5 (moderate deformity), or 1 (severe deformity), with a maximum possible score of 6. The score was divided into 2 subtotals of 3 points each: the midfoot (medial crease, curvature of the lateral border, and lateral part of the head of the talus) and hindfoot (posterior crease, empty heel, and rigid equinus).^[28,29]^ The need for PAT was independently and blindly assessed by 3 doctors trained in the application of the Pirani scale for each foot. Likewise, age (measured in months) and sex (female and male) were obtained from parents and medical records. Height (cm), weight (kg), and body maximum index (BMI) (kg/m^2^) were obtained as follows: length for children who could not stand being taken with a pediatric length board with a fixed headpiece and adjustable footpiece on a hard level surface, 2 health care professionals were required to accurately obtain the measurements. Children >2 years of age who could not stand, recumbent length was taken (length ‐ 0.7 cm = height), children who could stand a pediatric stadiometer on level ground and secure to the wall were used; in children <2 years of age, standing, we used standing height (length + 0.7 cm = height), both according to the Frankfort horizontal plane, to weight children a digital scale calibrated in a flat hard surface was used; if the child was unable to independently stan the health care giver weight themselves, then re-weight while holding the child and subtracting the weights to obtain the differences.^[30]^ BMI for Birth to 2 years was based on the formula (length + 0.7 cm); children >2 years old, in the formula (length ‐ 0.7 cm), and finally weight in kilograms/[height in meters × height in meters].^[31]^ Similarly, the number of plaster casts, affected extremities, recurrences, and complications were collected. Pre- and postoperative dorsal flexion of the ankle were measured using a manual goniometer (the fixed arm was placed at the peroneal malleolus vertex level, and the moving arm was aligned parallel to the fifth metatarsal, with the patient lying supine on their back and feet freely outside the bed). Postoperative measurements were also performed.^[32]^ Independent measurements were performed twice, and the average was taken as the final measurement. The day before PAT, a US review of the AT was conducted to measure the anteroposterior length, transverse diameter, and circumference in mm, as well as the area in mm^2^. The measurements were performed twice to obtain the average of the definitive measurements. Recurrence was diagnosed by the treating orthopedic doctor in the presence of an equinus, varus in the hindfoot, an adductus of the midfoot with dynamic supination, or support on the lateral bar of the foot.^[33]^

### 2.3. Description of the index tests

Tenotomy was scheduled in the operating room using the standard PAT technique under sedation. To enhance tendon palpation, local subcutaneous anesthesia (1 mL of 5% lidocaine) was applied immediately after the incision. A #15 scalpel blade was used on a marked area 1 cm from the tendon insertion, perpendicular to the foot in the coronal plane, and parallel to the fibers of the tendo-achilles. The scalpel was advanced slowly and carefully to divide the tensioned tendo-achilles after probing the area with the blade tip. The blade was rotated 90° and translated posteriorly to complete the procedure.^[34]^ Then, with the ankle in dorsiflexion forced, the sensation of “click” or “pop” was registered (a sensation of cutting a tense tendon, which is felt only once by the person performing the initial incision). Pediatric orthopedic specialists with at least 5 years of experience and training in the Ponseti method and PAT performed the incision and classified it as present or absent. To confirm this result, US was used as a standard reference (see below).

The Thompson test (absence of plantar flexion upon pressing the gastrocnemius muscle) was performed twice, once the foot was outside the surgery table,^[35]^ and the sedation effect was relieved in a blinded and independent manner by 2 pediatric orthopedic surgeons with more than 5 years of experience. The wound was then covered, followed by the placement of a final plaster cast using the method described by Ponseti,^[23]^ in a position corrected for maximum dorsiflexion possible (10–20°) and abduction of 70°, and left for 4 weeks. Acetaminophen was prescribed orally at a dose of 15 mg/kg body weight, and after recovering from anesthesia, the patient was discharged and fed a diet. The PAT and plaster cast placement were performed by an orthopedic surgeon certified using the Ponseti method.

### 2.4. Description of the reference standard

**Figure 1.**
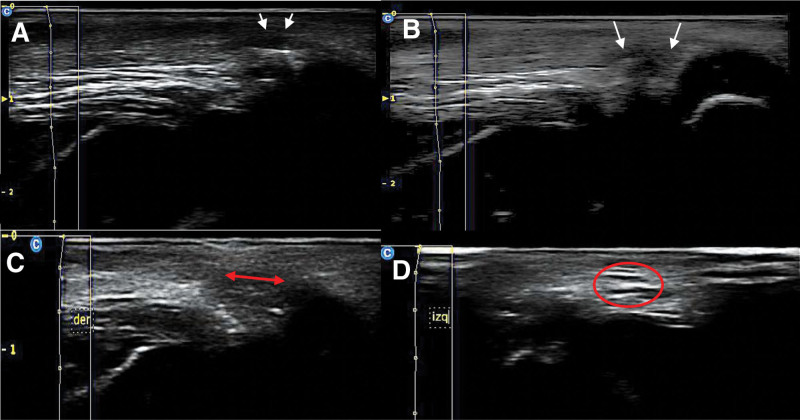
Sagittal image of an incomplete tendon section revealing reverberation and textured echoes with residual fibers after an incomplete tendon cut (A and B). The sagittal view also highlights a hematoma, tendon retraction, and a visible gap (C). Moreover, the axial view shows a lack of achilles tendons (D).

To confirm the AT cutoff, the US^[12]^ system CHISON ECO 6 Digital Color Doppler (Chison Medical Technologies Co., Ltd., Jiangsu, China) and a linear array transducer L7M-A (5.3–10.0 MHz) were used. Two musculoskeletal ultrasound-trained radiologists, blinded and independent of Thompson sign exploration and “click” or “pop” recording, performed US 10 mm from the AT insertion site employing a sterile bag to cover the transducer and using in the site of PAT an insulin needle, which served as contrast, the patient was placed on the operating table with her knees in extension and flexion, and a cut or complete tear of the tendon was evaluated and confirmed (Fig. 1). In the event of disagreement in the assessment, a third musculoskeletal ultrasound-trained radiologist was consulted to confirm the visualization of the cut, which was considered complete or incomplete. In the case of an incomplete tendon cut, the surgeon was instructed to perform a new cut using the same technique, which was then confirmed with US to be a complete tendon cut. These were considered false positives in the analysis.

### 2.5. Statistical methods

Qualitative variables were expressed as frequencies and percentages, whereas quantitative variables were represented as means or medians. The distribution of quantitative variables was estimated using the Kolmogorov–Smirnov test. Pearson chi-square test was used to compare the frequencies and percentages of dichotomous qualitative variables. Interobserver reliability was assessed using the intraclass correlation coefficient and Cohen kappa coefficient. To estimate the Se and Sp, the positive predictive value, negative predictive value, diagnostic accuracy, positive likelihood ratio, negative likelihood ratio, posttest probability, Fagan nomogram, free programs (www.medcalc.org/calc/diagnostic\_test.php), and (araw.mede.uic.edu/cgi-bin/testcalc.pl) were used. Spearman rank correlation was used to evaluate the associations between the number of plaster casts and patient age and the final Pirani score. To predict the risk of recurrence based on the final Pirani score, receiver operating characteristic (ROC) curve analysis was performed, along with an analysis of the area under the curve (AUC). Statistical significance was set at *P* < .05. All statistical analyses were performed using SPSS version 28 (SPSS, Armonk, NY).

## 3. Results

Forty-five feet of 30 patients with ICTEV who met the inclusion criteria were analyzed (flowchart). There were 15 (50%) bilateral patients and 9 (30%) right ICTEV patients. The median age was 11 months, with an interquartile range (IQR) of 4–6 months and a range (R) of 1–60 months. Eighteen (60%) patients were male. Weight and BMI means was 18.5 kg/m^2^ (SD 18) and 8.8 kg (SD 3.1) respectively. Median for height was 0.67 m, interquartile range (IQR) 0.60–0.73 m. The median number of plaster casts applied was 11, IQR = 11–15. The mean preoperative equino-plantar flexion angle was ‐31.2° (SD 12.5°), the median postoperative dorsal flexion angle was 10°, the IQR = 10–20°, and the range (R) was ‐10 to 30°. No arterial, venous, or nervous lesions or infections were detected. There were 9 (20%) recurrences, and 2 patients did not achieve neutrality at ‐5° and ‐10°.

From the measurements taken in centimeters and expressed as the means and SD in the AT, the anteroposterior length was 0.23 (0.04), the transverse length was 0.43 (0.1), and the circumference was a diameter of 1.25 (0.27). The area obtained a median of 0.08, with an IQR = 0.06–0.11 cm^2^.

The final score on the Pirani scale had a median of 2.5 IQR = 2–3.5. The interobserver reliability of the final measurements of the total Pirani score showed an intraclass correlation coefficient (0.92; 95% CI = 0.88 − 0.96; *P* < .001), hindfoot (0.75; 95% CI = 0.59 − 0.85; *P* < .001), (0.79; 95% CI = 0.66 − 0.88; *P* < .001) for the midfoot. Thompson sign achieved an interobserver reliability of κ = 0.83. The final Pirani scale score and number of plaster casts were correlated (*rho* = 0.36, 95% CI = 0.07 − 0.60; *P* = .014). The number of plaster casts and patient age were correlated (*rho* = 0.41; 95% CI = 0.12 − 0.63; *P* = .005). Adhesion to the orthosis and risk with respect to recurrence showed differences (88.9 vs 11.1; χ^2^ = 16.23; *P* < .001). The number of plaster casts and risk of recurrence were not significantly different (*Z* = ‐1.05; *P* = .3).

**Figure 2.**
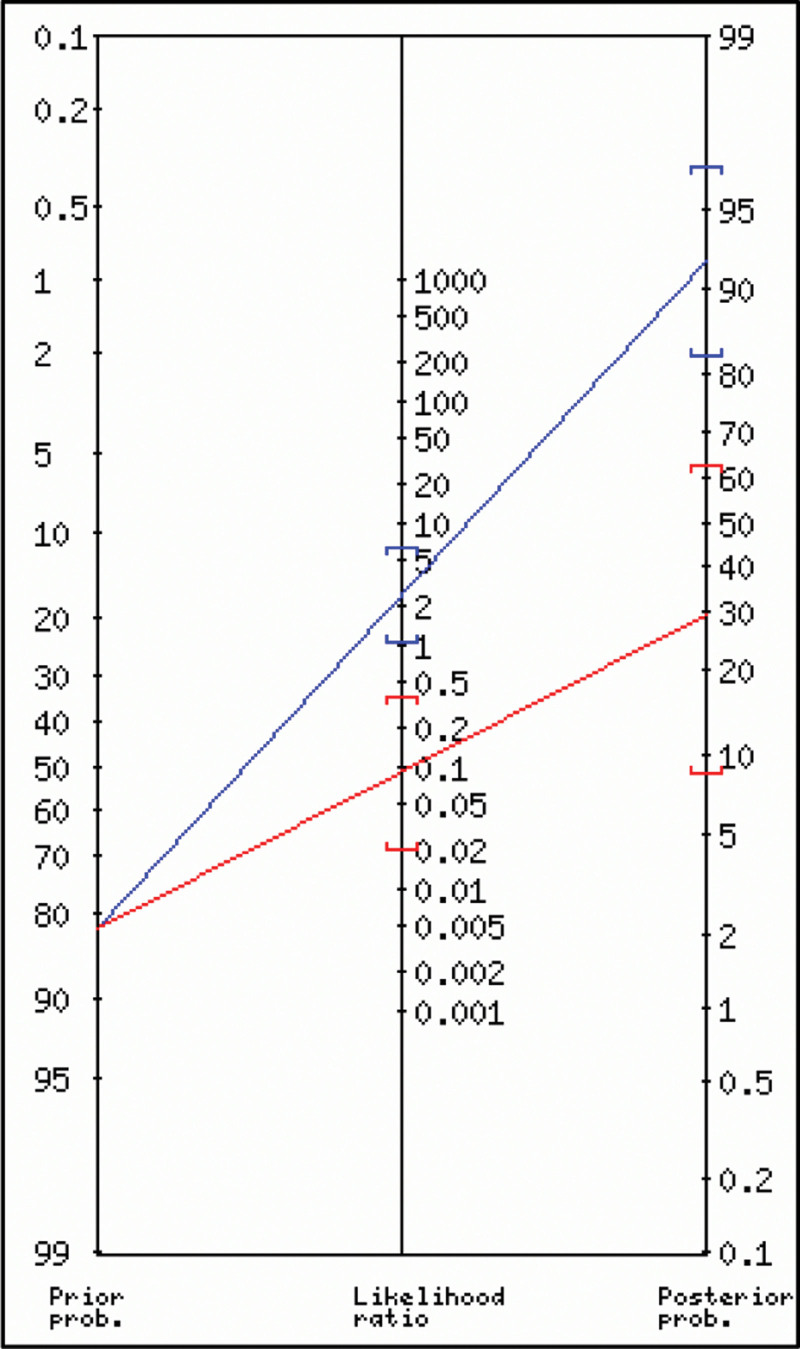
Fagan nomogram for the analysis of the posttest probability of positive and negative “click” or “pop” sensations. with a pretest probability of 82%. NLR = negative likelihood ratio, PLR = positive likelihood ratio, PP = posterior probability (odds).

**Figure 3.**
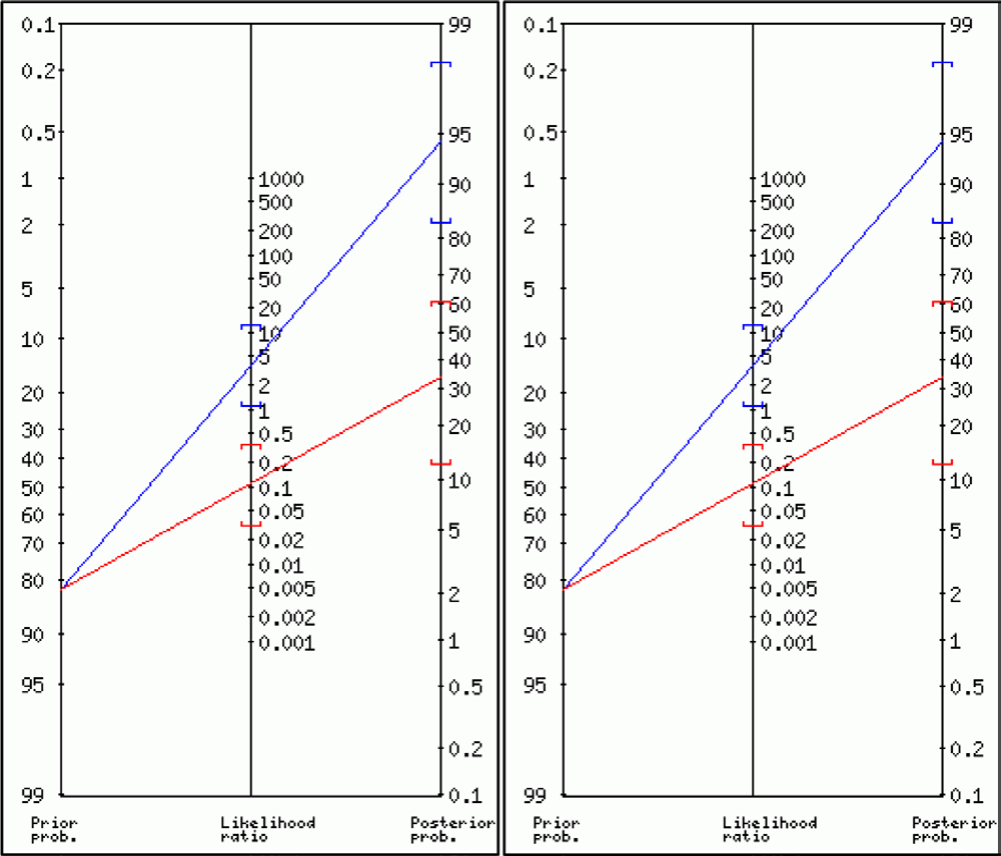
Fagan nomogram for posttest probability analysis of positive and negative Thompson signs in the 2 evaluations. PLR = positive likelihood ratio, PP = posterior probability (odds), NLR = negative likelihood ratio.

**Figure 4.**
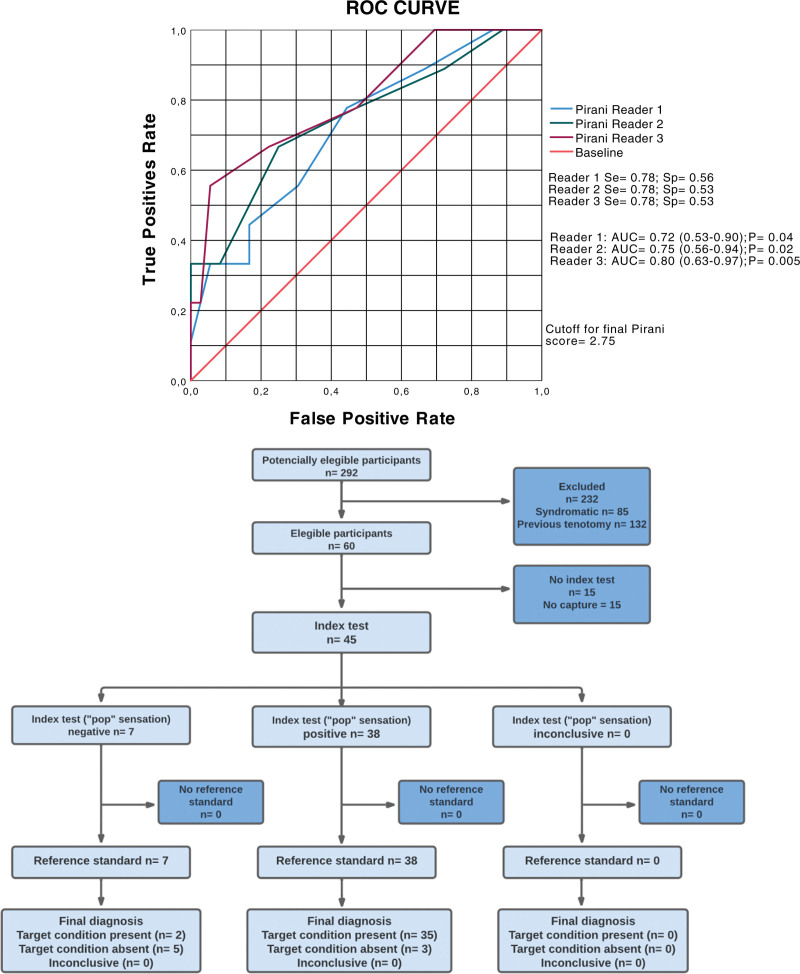
Shows the results of the predictive analysis of the 3 ROC curves, the area under the curve (AUC) of the final score on the Pirani scale, and the risk of recurrence after achilles tenotomy at the same cutoff point in the 3 evaluations.

A total of 38 chances (84%) were reported by the surgeon to have experienced the sensation of a “click” or “pop,” while the absence of plantar flexion (Thompson sign) in 2 evaluations was positive in 33 and 36 patients (73% and 80%, respectively). Complete US tear confirmation was observed in 37 (82 %) patients. The estimation of the “click” or “pop” sensation versus US yielded a Se = 0.95 and Sp = 0.63, while the estimation of the Thompson sign versus US in 2 evaluations resulted in a Se = 0.87 and 0.92 and Sp = 0.88 and 0.75, respectively (Table 1). The posttest probability of the “click” or “pop” sign was 87% (Fig. 2), and that of the Thompson sign was 94% and 97%, respectively, in the 2 evaluations (Fig. 3). The ROC curve to determine the risk of recurrence in relation to the final Pirani score obtained the best cutoff point of 2.75 for all 3 Pirani measurements (Fig. 4). The ROC curve only for the hindfoot component had an AUC of 0.77, a cutoff point of 1.75, and Se and Sp values of 0.77 and 0.59, respectively. The combined estimates of the “click” or “pop” signs plus the Thompson signs from the first evaluation yielded Se = 0.99 and Sp = 0.47, and the Thompson signs from the second evaluation produced Se = 0.99 and Sp = 0.55.

**Table 1:** Compares the sensation of a “click” or “pop” and the Thompson sign in 2 evaluations via ultrasonographic analysis.

	Pop sign	Thompson (reader 1)	Thompson (reader 2)
Sensitivity	0.95	0.87	0.92
Specificity	0.63	0.88	0.75
PLR	2.52	6.92	3.68
NLR	0.09	0.15	0.11
Prevalence	82.2	82.2	82.2
PPV	92.1	96.9	94.4
NPV	71.4	58.3	66.7
Accuracy	88.9	86.7	88.9

NLR = negative likelihood ratio, NPV = negative predictive value, PLR = positive likelihood ratio, PPV = positive predictive value.

## 4. Discussion

The finding of relevance in this study was that surgeons reported a sensation of “pop” or “click” upon performing PAT in 84% of the patients, whereas 82% of the complete cuts were obtained with US, with a Se = 0.95. This finding contrasts with the results obtained by Karami,^[16]^ who reported 81% with the sensation of “pop” or “click” versus 44% with a complete cut of the AT by US. This difference could be explained by differences in patient age. In our study, the median age was 11 months, which was greater than that reported by Karami et al In patients with small feet, the possibility of damaging nearby structures may inadvertently modify the technique, predisposing them to an incomplete tendon cut,^[15]^ as it is most likely that a complete cut of the AT will be obtained in patients 3 months or older.^[36]^ Another difference from the Karami study may be due to local anesthetic infiltration before PAT, which may obscure palpation of the AT and predispose it to incomplete cutting.^[7]^ Similarly, complete AT release can sometimes be hindered by the rigidity of the posterior capsule, which predisposes patients to incomplete release because full dorsiflexion after tenotomy cannot be achieved. Although Grigoriou^[4]^ did not report any difference between open tenotomy plus capsulotomy and PAT alone, we believe that in cases where neutral dorsiflexion cannot be achieved, it would be beneficial to perform open tenotomy along with posterior capsulotomy to reduce the risk of recurrence and damage to vascular or neural structures owing to repetitive movements in the PAT. However, this requires further investigation.

Various aspects must be considered in this report, including the Thompson sign, as a potential indicator of complete transection of the AT after percutaneous tenotomy. In separate evaluations, Thompson sign showed a Se values of 0.92 and 0.87, which is consistent with the findings of Maranho report,^[37]^ who stated that the Thompson sign is sensitive for the detection of residual tendinous fibers in incomplete tenotomy and that when the sign is negative and is evaluated by US, it confirms partial tenotomy. However, the Se and Sp values were not estimated in that study. Additionally, in other reports where US was not used to confirm tendon cutting but the Thompson maneuver was performed, it was reported that the signs were consistent in confirming complete tendon cutting. In these reports, only 4 cases of recurrence were presented, indicating that the Thompson sign is a reliable indicator of complete tendon cutting, along with dorsiflexion movement and palpable depression of the breach between the tendon nodes, which indicates that clinical signs are reliable in confirming tendon cutting.^[38–40]^ The following sentence suggests that US can be avoided in most cases of PAT performed, whether in the operating room or outpatient setting. This finding is supported by Kumar report,^[41]^ which states that US-guided PAT does not have any additional advantages over conventional PAT, as confirmed clinically, and that there are no differences in long-term results.

Furthermore, the ability of the final Pirani score to predict recurrence risk in this report was fair, with a cutoff of 2.75 and AUCs ranging from 0.72 to 0.80 and Se = 0.78 and Sp = 0.56. This predictive capacity has been estimated, but for the initial Pirani score in a similar study, with Se = 0.92 and Sp = 0.68.^[42]^ The final score was evaluated because the components of the hindfoot could be associated with the risk of recurrence, as demonstrated by the ROC curve, which showed that the hindfoot score was an independent predictor of the risk of recurrence. Additionally, a report comparing initial Pirani scores between patients with and without recurrence revealed that patients with high initial hindfoot scores had a greater frequency of recurrence.^[20]^ Thus, the initial or final component of the hindfoot should be considered an independent predictor of recurrence.

Moreover, the correlation between the number of plaster casts and the final Pirani score in our study was weak compared to that of Lampasi,^[43]^ who obtained a moderate correlation between the same components but with the initial Pirani score. Therefore, the Pirani score is a regular indicator of the number of plaster casts. Similarly, in this study, a weak correlation was observed between patient age and number of plaster casts, which is consistent with the findings of Agarwal.^[44]^ Perhaps factors such as patient care and removal of the cast by the child’s caregiver the day before could predispose the child to recurrence of the deformity and a greater number of casts. Additionally, a meta-analysis^[45]^ reported inconsistent results on the probability of recurrence for both the number of plaster casts and patient age, indicating that these factors do not play a decisive role in recurrence, as does the limited muscle activity of eversion and lack of attachment to the orthosis.

The strengths of this study lie in the calculation of the sample size, which allowed the observation of small differences. Similarly, the PAT was performed by different groups of expert surgeons during Ponseti treatment, suggesting variability in the analysis of the “pop” or “click” signs, which can be obtained only once. The Thompson sign was also estimated to be a clinical sign for confirmation of the tendon cut, and the interobserver variability was excellent. Additionally, the study data were prolective obtained to avoid bias from nonresponse or incomplete clinical records.

The limitations of the study include the inclusion of patients older than 6 months of age, which may have made it easier to perform percutaneous tendon release by palpating it clearly. Additionally, all patients were sedated and controlled in the operating room, which provided comfort for performing tenotomy and US analysis by preventing irritation and defensive movements that occur in the ambulatory environment, which may have led to a higher rate of complete release. Another disadvantage was the lack of follow-up beyond 6 months, which could have caused underreporting of recurrences.

In conclusion, the “pop” or “click” signs and Thompson signs, which indicate a complete tear of the AT, have high accuracy, prevalence, and posttest probability, and together enhance the confirmation of the percutaneous tendon cut. The correlation between the number of plaster casts and patient age is weak. The ability of the final total Pirani and hindfoot scores to predict recurrence risk was fair.

## Author contributions

**Conceptualization:** Sergio Charles-Lozoya.

**Data curation:** Jorge Luis Alvarado-Alanis, Miguel Leonardo de la Parra-Márquez, Arnoldo Salas-Delgado, Marcela Araceli Segoviano-Mendoza.

**Formal analysis:** Sergio Charles-Lozoya, Héctor Cobos-Aguilar, Jorge Luis Alvarado-Alanis, Arnoldo Salas-Delgado, Marcela Araceli Segoviano-Mendoza.

**Methodology:** Héctor Cobos-Aguilar, Miguel Leonardo de la Parra-Márquez.

**Supervision:** Sergio Charles-Lozoya, Héctor Eliud Arriaga-Cazares, Jocelyn Verónica Montes-Cruz.

**Validation:** Sergio Charles-Lozoya, Héctor Cobos-Aguilar, Jorge Luis Alvarado-Alanis, Arnoldo Salas-Delgado, Marcela Araceli Segoviano-Mendoza.

**Writing – original draft:** Sergio Charles-Lozoya.

**Writing – review & editing:** Sergio Charles-Lozoya, Héctor Cobos-Aguilar, Jorge Luis Alvarado-Alanis, Arnoldo Salas-Delgado, Marcela Araceli Segoviano-Mendoza.
